# Multidrug-resistant *Klebsiella pneumoniae* and *Klebsiella oxytoca* isolated from backyard broiler chickens and their contacts with antimicrobial resistance genes of *Klebsiella pneumoniae*

**DOI:** 10.1038/s41429-025-00875-y

**Published:** 2025-11-20

**Authors:** Shimaa El Baz, Abdelfattah H. Eladl, Reham A. El-Shafei, Mohamed A. Elmorsy

**Affiliations:** 1 Department of Hygiene and Zoonoses, Faculty of Veterinary Medicine, Mansoura University, Mansoura, Egypt; 2 Department of Poultry and Rabbit Diseases, Faculty of Veterinary Medicine, Mansoura University, Mansoura, Egypt; 3 Department of Pharmacology, Faculty of Veterinary Medicine, Mansoura University, Mansoura, Egypt

**Keywords:** Diseases, Bacteriology

## Abstract

The objective of this study was to investigate the prevalence and phenotypic identification of antimicrobial resistance of *K. pneumoniae* and *K. oxytoca* recovered from backyard broiler chickens and their human contacts. The serotypes and genotypes of antibiotic resistance genes of *K. pneumoniae* isolates were investigated. A total of 80 samples were collected from backyard broiler chickens that showed signs of illness, sneezing, coughing, and diarrhea. Twenty stool samples were collected from individuals who had been in contact simultaneously. In total, 19 *Klebsiella* species were isolated, 12 of which were from broiler chicken samples and seven from human stool samples. Recovery rates of *K. pneumoniae* were 11.3% (*n *= 9/80) and 15% (*n* = 3/20) of broiler and human stool samples, respectively. *K. oxytoca* was detected in 3 of 80 (3.75%) broiler chicken and 4 of 20 (20%) human stool samples. Antimicrobial susceptibility showed that all 19 *Klebsiella* isolates were resistant to erythromycin and clindamycin (100%), followed by penicillin (94.7%) and ampicillin (84.3%). Within the 12 *K. pneumoniae* isolates, the most common serotype was K1. Antibiotic resistance gene profile of *K. pneumoniae* isolates was observed, with a high carrying rate of the macrolide gene *ermB* (91.7%), followed by the β-lactam genes *bla*_*TEM*_ (75%) and *bla*_*CTX-M1*_ (66.7%). In conclusion, the emergence of multidrug-resistant (MDR) bacteria, *K. pneumoniae* and *K. oxytoca* in broiler backyard chickens and their human contacts is alarming, particularly for erythromycin and clindamycin. This underscores the need for alternatives like vaccination, probiotics, and better biosecurity to combat antimicrobial resistance.

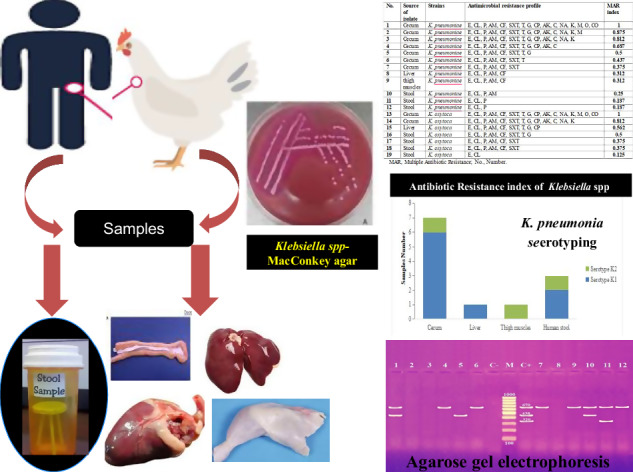

## Introduction

*Klebsiella* is a member of the Enterobacteriaceae family, one of the largest groups of Gram-negative bacteria [1]. The species of greatest clinical importance within this genus are *K. pneumoniae* and *K. oxytoca*, which are opportunistic pathogens responsible for serious diseases in both humans and animals, raising public health concerns [2]. The most important reason why this pathogen is of great concern is the problem of antimicrobial resistance that hampers efforts to control it. *K. pneumoniae* is primarily found in the oropharynx and is associated with blood, urinary tract, and respiratory tract infections in humans [3]. It is a major cause of hospital-acquired infections and actively produces enzymes that confer resistance to β-lactam antibiotics [4].

One of the important pathogens in poultry farming that has emerged is *K. pneumoniae*, which mainly causes respiratory symptoms in broiler chickens, which can lead to high mortality rates and significant economic losses. The isolation rate of this bacterium has been reported to range from 9% to 35% [5]. Some factors of *Klebsiella* pathogenicity have been proposed, namely the smooth lipopolysaccharide (LPS) antigen (O antigen) and the capsular polysaccharide antigen (K antigen, the most important virulence factor in *K. pneumoniae*) [6]. Additionally, fimbrial adhesion proteins facilitate the adherence of bacterial cells to host tissues [2].

In poultry farming, antibiotics are often used not only as medicines but also as prophylactic agents and growth promoters [7]. As a result, *Klebsiella* - a notable zoonotic pathogen- frequently exhibits multidrug-resistant (MDR). This leads to increased growth of super-resistant bacterial strains and the risk of foodborne diseases that are transmitted to humans through the food chain [8]. The emergence of MDR bacteria poses significant therapeutic challenges worldwide, particularly in the treatment of infections caused by *Klebsiella* species, especially *K. pneumoniae*, due to its ability to acquire and disseminate resistance genes [9]. *K. pneumoniae* commonly harbors multidrug resistance (MDR) determinants, including plasmid-mediated quinolone resistance (PMQR) and extended-spectrum β-lactamase (ESBL) genes, most notably blaOXA, blaCTX, and blaTEM which confer substantial resistance to β-lactam antibiotics [10, 11]. The first line of treatment for serious infections caused by *K. pneumoniae* that produces ESBLs is carbapenems. As the most common bacteria associated with *K. pneumoniae* carbapenemase (KPC) resistance determinants, carbapenem-resistant *K. pneumoniae* strains have emerged [12].

The zoonotic potential of *K. pneumoniae* has been emphasized, for example, with research highlighting its presence in food animals and retail meat, and linking it to human infection through direct contact and consumption. This trend indicates that these bacteria could be transmitted from animals to humans through animal contact and food consumption, posing a major public health risk [13]. Therefore, it is important to conduct regular monitoring and surveillance of resistant bacteria in foods to implement appropriate control strategies.

The two main types of poultry production – intensive (commercial) and extensive (backyard) – are used all over the world, including Egypt. *Klebsiella* has emerged as a growing problem for poultry and humans worldwide. Despite the increasing prevalence of *Klebsiella* species in the food chain, research on *K. pneumoniae* and *K. oxytoca* in backyard broiler chickens and the risk of human infection through animal contact or food consumption remains limited worldwide, including in Egypt. Therefore, this study aimed to evaluate the prevalence and pattern of antibiotic resistance of these bacteria in backyard broiler chickens and humans who have contact with those chickens.

## Materials and methods

### Ethical approval

This study was approved by the Ethical Committee of the Faculty of Veterinary Medicine, Mansoura University, Egypt, and was conducted in accordance with the guidelines of the “Guide for the Care and Use of Laboratory Animals” with approval number: MU-ACUC (VM.R.25.02.213).

### Samples collection

Birds were collected from different owners in urban areas at different localities in Dakahlia, Gharbia and Damietta Governorates in Egypt. Samples were collected over an 8-month period, from February to September 2024. The birds were Fayoumi and White Baladi breeds raised in backyards. A total of 80 broiler chicken samples from cecum, liver, heart, and thigh muscles (20 each) were collected from backyard broiler carcasses, aged 35–50 days. The chickens showed signs of illness, including loss of appetite, sneezing, coughing, and diarrhea. At the same time, 20 stool samples were collected from individuals who had been in contact with these chickens. All collected samples were sent to the laboratory for bacteriological examination in iceboxes at 4 °C.

### Samples preparation

Samples were prepared as described previously [14]. Briefly, samples (25 g) were then placed in a sterile mortar, with 225 mL of buffered peptone water (Oxoid Ltd., Basingstoke, UK; BPW; CM0509B) and homogenized for 2 min. The homogenates were then transferred to sterile tubes and incubated at 37 °C overnight.

### Isolation and identification of *Klebsiella* species

A loopful of enriched BPW was placed on MacConkey agar plates (M0007B, Oxoid Ltd., Basingstoke, UK) and incubated aerobically overnight at 37 °C [15]. Mucoid and pink colonies were then picked and subcultured on MacConkey agar (Oxoid Ltd., Basingstoke, UK). The suspected pure *Klebsiella* species were identified by the following biochemical tests: Indole, methyl red, Voges-Proskauer and citrate utilization (IMViC) tests, hydrogen sulfide production test, urease test, gelatin hydrolysis test, nitrate reduction test, ornithine decarboxylase (ODC) detection, L-lysine decarboxylase (LDC) detection, arginine decarboxylase (ADH) detection, β-galactosidase (ONPG) detection, and fermentation of sugars including glucose, sucrose, lactose, maltose and mannitol [16].

### String test

In order to better understand the virulence mechanisms of the hypermucoviscous (HMV) and as part of the culture characterization process, phenotype string tests of *K. pneumoniae* were also performed to assess microviscosity [17]. In the hypermucoviscous *K. pneumonia* (hvKp) string test, a viscous strings longer than 5 mm when the colony contacted by a loop and then stretched vertically from the surface of the agar plate identified as a positive mucoid phenotype [18].

### Serological identification of capsular antigen

Serological testing for the two antigenic types (K1 and K2) of *K. pneumoniae* was performed using the Quellung test “Neufeld reaction” as previously described [19]. The Quellung test was performed according to the manufacturer’s protocol, which included microscopic observation of the interaction between antigens and antibodies. A bacterial suspension (equivalent to 1.5 × 10^8^ CFU/mL) was prepared and mixed well with antiserum targeting the K1 and K2 capsular antigens of *K. pneumoniae* (Staten Serum Institute, Copenhagen, Denmark) on a glass slide. Microscopically, a positive Quellung test reaction is characterized by a clearly defined halo capsule surrounding the dark blue-stained bacterial cell.

### Antimicrobial susceptibility testing

Phenotypic and genetic identification of *Klebsiella* species was performed at the Food Analysis Center, Faculty of Veterinary Medicine, Benha University, Egypt, according to the guidelines described by the Clinical and Laboratory Standards Institute [20]. The antimicrobial susceptibility test was conducted by the agar disk diffusion method against 16 agents of seven categories of clinically relevant and commonly used antibiotics. The study included 19 *Klebsiell*a species isolates, comprising 12 isolates (*K. pneumonia* = 9 and *K. oxytoca* = 4) from broiler chickens and seven (*K. pneumonia* = 4 and *K. oxytoca* = 3) from human stool samples. The following antibiotic disks were used: erythromycin (E; 15 μg), clindamycin (CL; 10 μg), penicillin (P; 10 IU), ampicillin (AM; 10 μg), cefotaxime (CF; 30 μg), sulfamethoxazole (SXT; 25 μg), tetracycline (T; 30 μg), gentamicin (G; 10 μg), ciprofloxacin (CP; 5 μg), amikacin (AK; 30 μg), chloramphenicol (C; 30 μg), nalidixic acid (NA; 30 μg), kanamycin (K; 30 μg), meropenem (M; 10 μg), ofloxacin (O; 5 μg), and colistin (CO; 25 μg). A single colony of the tested bacterial strain was picked and spread evenly on the surface of Mueller–Hinton agar (Basingstoke, UK, Oxoid Ltd; CM0337). Antibiotic discs were placed on the inoculated plates, and then incubated at 37 °C for 16–18 h. After incubation, the diameters of the inhibition zone were measured using a micrometer and classified into sensitive, intermediate, and resistant. The susceptibility of *Klebsiell*a species isolates to the tested antimicrobial panels was evaluated according to the guidelines described by the Clinical and Laboratory Standards Institute [20]. The following formula was used to determine the multiple antibiotic resistance (MAR) index for each strain: MAR index = number of resistances/total number of antibiotics tested. If a strain shows resistance to one of three or more antimicrobial categories, it is considered resistant. *Escherichia coli* (ATCC 25922) and *K. pneumonia*e (ATCC 700603) strains were used as quality control strains throughout the study for both culture and antimicrobial susceptibility testing.

### Screening of *K. pneumoniae* for antimicrobial-resistance (AMR) genes

#### DNA extraction

A single colony of the bacterial strains was incubated overnight in 100 µL of sterile distilled water. Cellular remnants were removed by centrifugation at 12,000 × *g* for 5 min after cell lysis by heating to 96 °C for 15 min. The resulting supernatant was used as a DNA template for PCR amplification and stored at −20 °C until further use [21]. Only *K. pneumonia* strains were screened for antimicrobial resistance genes.

#### Detection of antimicrobial resistance genes by polymerase chain reaction (PCR)

The amplification process was performed using a thermal cycler (Master Cycler, Eppendorf, Hamburg, Germany). The sequences, product sizes, and annealing temperatures for all forward and reverse primers used are detailed in Table 1 [14, 22–27]. All primers were obtained from Promega (Promega Corporation, Madison, USA) and were used at a concentration of 10 pmol/μl for both forward and reverse primers.

**Table 1:** Primer sequences for genotypic identification of antibiotic resistance genes of *K. pneumonia*

Antibiotic class	Target gene	Oligonucleotide sequence (5′ → 3′)	Product size (bp)	Annealing temperature	References
	*tetA*	F: 5′ GTAATTCTGAGCACTGTCGC ′3	965	54 °C for 1 min	[22]
Tetracycline		R: 5′ CTGCCTGGACAACATTGCTT ′3			
Quinolones	*gyrA*	F: 5′ CGACCTTGCGAGAGAAAT ′3	626	54 °C for 1 min	[23]
		R: 5′ GTTCCATCAGCCCTTCAA ′3			
	*bla*_*TEM*_	F: 5′ ATCAGCAATAAACCAGC ′3	516	54 °C for 1 min	[14]
β-lactam		R: 5′ CCCCGAAGAACGTTTTC ′3			
	*bla*_*CTX-M1*_	F: 5′ AAGACTGGGTGTGGCATTGA ′3	670	61 °C for 35 s	[24]
		R: 5′ AGGCTGGGTGAAGTAAGTGA ′3			
Sulfonamides	*sul1*	F: 5′ TCACCGAGGACTCCTTCTTC ′3	331	54 °C for 1 min	[25]
		R: 5′ CAGTCCGCCTCAGCAATATC ′3			
Carbapenems	*bla*_*OXA-48*_	F: 5′ GCGTGGTTAAGGATGAACAC ′3	438	61 °C for 35 s	[26]
		R: 5′ CATCAAGTTCAACCCAACCG ′3			
Peptides	*mcr-1*	F: 5′ AGTCCGTTTGTTCTTGTGGC ′3	320	58 °C for 90 s	[27]
		R: 5′ AGATCCTTGGTCTCGGCTTG ′3			
*Macrolides*	*ermB*	F**: 5′** GAAAAGGTACTCAACCAAATA ′3	639	53 °C for 30 s	[14]
		R: 5′ GTAACGGTACTTAAATTGTTTAC ′3			

##### To detect resistance genes, three PCR reactions were performed, consisting of two multiplex reactions and one uniplex PCR reaction

The first multiplex was used to amplify the genes *tetA*, *gyrA*, *bla*_*TEM*_, and *sul1*. Each 50 µL PCR reaction contains the following components: 25 μL of RedMasterMix (2×) Taq PCR (GENAXXON Bioscience, Ulm, Germany), 3 μL of DNA template, 2 μL of forward primers, and 2 μL of reverse primers. A total volume of 50 μl was obtained by adding nuclease-free water. Multiplex PCR conditions were performed using the procedures previously outlined [14]. The amplification process started with a pre-denaturation step at 94 °C for 2 min. The following steps were included in each of the 30 cycles: 30 s denaturation at 94 °C, 1 min of annealing at 54 °C, and 4 min of extension at 72 °C. A final extension was performed for 10 min at 72 °C.

The second multiplex was used to amplify the *bla*_*CTX-M1*_, *bla*_*OXA-48*_, and *mcr-1*genes. Each 50 µL reaction was used using 5 µL of DNA solution, 25 µL of RedMasterMix (2×) Taq PCR (GENAXXON, Bioscience), and 2 µL of forward and reverse primers. A total volume of 50 μl was obtained by adding nuclease-free water. Multiplex PCR was performed according to protocols developed previously [28]. The cycle parameters were 10 min at 94 °C, followed by 30 cycles of 30 s at 94 °C, 35 s at 61 °C, and 1 min at 72 °C. Finally, the final extension step lasted 9 min at 72 °C.

PCR reaction for amplification of the uniplex *ermB* gene consisted of 12.5 μL of My Taq™ HS Red Mix (Bioline, Meridian Life Science Inc, Ohio, United States), 1 μL of DNA extract, 1 μL of forward primer, 1 μL of reverse primer, and 9.5 μL of nuclease-free water. The PCR cycling conditions were performed according to the guidelines previously provided [29]. Thirty cycles of denaturation at 95 °C for 3 min, denaturation at 95 °C for 30 s, and annealing at 60 °C for 30 s were all part of the PCR cycling conditions. All PCR products were visualized by electrophoresis using 1% agarose gel (Puregene™, India). The gel was run at a constant voltage of 120 V for 1 h and then stained in 1 μg/mL ethidium bromide for 10 min. The gel was imaged under a gel document with ultraviolet illumination (acculab, Montreal, Quebec, Canada).

## Results

### Prevalence of *K. pneumoniae* and *K. oxytoca* in broiler chickens and human samples

*Klebsiella* species was isolated from 19 out of 100 (19%) of the total samples examined. Among these samples, *K. pneumoniae* was isolated from 12 out of 100 samples (12%) of the total samples, while *K. oxytoca* was positive in 7 out of 100 samples (7%; Table 2). Among the 12 positive *K. pneumoniae* isolates, 9 (11.3%) strains were recovered from broiler samples, of which 7/20 (35%), 1/20 (5%), and 1/20 (5%) were recovered from cecal, liver, and thigh muscle samples, respectively. Meanwhile, no *K*. *pneumoniae* was recovered from the heart samples. Regarding human stool samples, 3/20 (15%) of the samples contained *K. pneumoniae*. On the other hand, the total recovery from *K. oxytoca* was 3/80 (3.75%) of broiler samples, while 4/20 (20%) of human stool samples tested positive for *K. oxytoca*, as shown in Table 2. In chicken samples, *K. oxytoca* was recovered from 2/20 (10%) and 1/20 (5%) of bird liver and cecum, respectively (Table 2).

**Table 2:** Prevalence of *Klebsiella* species in broiler samples and human stool regarding to biochemical identification

	Samples source	Samples number	*Klebsiella* spp		*K. pneumoniae*		*K. pneumoniae String test*			*K. oxytoca*	
							hvKp		cKp		
			No.	%	No.	%	No.		No.	No.	%
Broiler chickens	Cecum	20	9	45	7	35	5		2	2	10
	Liver	20	2	10	1	5	0		1	1	5
	Heart	20	0	0	0	0	0		0	0	0
	Thigh muscles	20	1	5	1	5	0		1	0	0
	Total	80	12	15	9	11.3	5		4	3	3.75
Human	Stool	20	7	35	3	15	2		1	4	20
	Total	100	19	19	12	12	7	5		7	7

*HvKp* Hypervirulent *K. pneumoniae*, *cKp* Classical *K. pneumoniae*, *No.* Number

### *K. pneumoniae* isolates of string test

The results of the string test showed that out of 9 *K. pneumoniae* isolates obtained from broiler chickens, 5 were identified as hvKp, all of which were isolated from cecal samples. In contrast, 4 isolates were classified as classical *K. pneumoniae* (cKp): 2 from cecal samples, 1 from liver, and 1 from thigh muscle. Regarding human *K. pneumoniae* isolates, two were classified as hvKp, while one was identified as cKp as shown in Table 2.

### Serotyping of *K. pneumoniae*

**Fig. 1:**
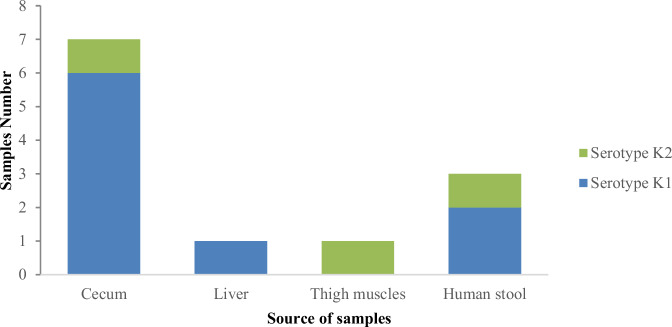
Serotype identification of *K. pneumoniae* strains recovered from broiler chicken samples and human stool. There was no isolates recovered from heart

Serotyping analysis revealed the K1 capsular types in 6/7, 1/1, and 2/3 of *K. pneumoniae* strains collected from broiler samples (cecum and liver) and stool samples, respectively. Meanwhile, the K2 capsular types were detected in 1/7 and 1/3 of *K. pneumoniae* strains obtained from broiler chickens’ cecum and human stool samples, respectively, as shown in Table 2 and Fig. 1.

### Phenotypic antimicrobial resistance of *K. pneumoniae* and *K. oxytoca* and MDR profiles

Nineteen samples tested positive for *Klebsiella* species against 16 antimicrobial agents from seven categories as shown in Table 3. The resistance rate to erythromycin and clindamycin was highest (100% each), followed by penicillin (94.7%) and ampicillin (84.2%). In contrast, the lowest resistance rate was observed for colistin and ofloxacin (10.5% each). Antimicrobial susceptibility profiling of *Klebsiella* species (*n* = 19) against 16 antimicrobial agents from seven different antibiotic classes revealed different patterns of antimicrobial resistance. It is worth noting that 100% (19/19) of *Klebsiella* isolates were resistant to at least two antimicrobials as shown in Table 4. Isolates obtained from broiler chickens showed a higher rate of MDR compared to isolates obtained from humans, as indicated in Table 4.

**Table 3:** Percentage of phenotypic identification of antibiotic resistance in *Klebsiella* species bacteria (*n* = 19) obtained from broiler chicken samples and human stool

Antibiotic classes	Antimicrobial agent	Susceptible		Intermediate		Resistance	
		No.	%	No.	%	No.	%
Macrolides	Erythromycin (E)	0	0	0	0	19	100
Lincosamide	Clindamycin (CL)	0	0	0	0	19	100
β-Lactam	Penicillin (P)	1	5.3	0	0	18	94.7
	Ampicillin (AM)	1	5.3	2	10.5	16	84.2
Cephalosporin	Cefotaxime (CF)	3	15.8	1	5.3	15	78.9
Sulfonamide	Sulphamethoxazol (SXT)	4	21.1	2	10.5	13	68.4
Tetracyclines	Tetracycline (T)	6	31.6	3	15.8	10	52.6
Aminoglycosides	Gentamicin (G)	7	36.8	3	15.8	9	47.4
	Amikacin (AK)	10	52.6	3	15.8	6	31.6
	Kanamycin (K)	14	73.7	0	0	5	26.3
Amphenicol-class antibacterial	Chloramphenicol (C)	12	63.2	1	5.3	6	31.6
Quinolone	Nalidixic acid (NA)	12	63.2	2	10.5	5	26.3
Carbapenem	Meropenem (M)	15	78.9	1	5.3	3	15.8
Fluorquinolones	Ciprofloxacin (CP)	11	57.9	1	5.3	7	36.8
	Ofloxacin (O)	16	84.2	1	5.3	2	10.5
Peptides	Colistin (CO)	17	89.5	0	0	2	10.5

**Table 4:** Multiple antibiotic resistance index of *Klebsiella* species isolates (*n *= 19) recovered from broiler chicken samples and human stool

No.	Source of isolate	Strains	Antimicrobial resistance profile	MAR index
1	Cecum	*K. pneumoniae*	E, CL, P, AM, CF, SXT, T, G, CP, AK, C, NA, K, M, O, CO	1
2	Cecum	*K. pneumoniae*	E, CL, P, AM, CF, SXT, T, G, CP, AK, C, NA, K, M	0.875
3	Cecum	*K. pneumoniae*	E, CL, P, AM, CF, SXT, T, G, CP, AK, C, NA, K	0.812
4	Cecum	*K. pneumoniae*	E, CL, P, AM, CF, SXT, T, G, CP, AK, C	0.687
5	Cecum	*K. pneumoniae*	E, CL, P, AM, CF, SXT, T, G	0.5
6	Cecum	*K. pneumoniae*	E, CL, P, AM, CF, SXT, T	0.437
7	Cecum	*K. pneumoniae*	E, CL, P, AM, CF, SXT	0.375
8	Liver	*K. pneumoniae*	E, CL, P, AM, CF	0.312
9	thigh muscles	*K. pneumoniae*	E, CL, P, AM, CF	0.312
10	Stool	*K. pneumoniae*	E, CL, P, AM	0.25
11	Stool	*K. pneumoniae*	E, CL, P	0.187
12	Stool	*K. pneumoniae*	E, CL, P	0.187
13	Cecum	*K. oxytoca*	E, CL, P, AM, CF, SXT, T, G, CP, AK, C, NA, K, M, O, CO	1
14	Cecum	*K. oxytoca*	E, CL, P, AM, CF, SXT, T, G, CP, AK, C, NA, K	0.812
15	Liver	*K. oxytoca*	E, CL, P, AM, CF, SXT, T, G, CP	0.562
16	Stool	*K. oxytoca*	E, CL, P, AM, CF, SXT, T, G	0.5
17	Stool	*K. oxytoca*	E, CL, P, AM, CF, SXT	0.375
18	Stool	*K. oxytoca*	E, CL, P, AM, CF, SXT	0.375
19	Stool	*K. oxytoca*	E, CL	0.125

*MAR* Multiple Antibiotic Resistance, *No.* Number

### Antibiotic resistance profile and multiple antibiotic resistance (MAR) index

The antibiotic resistance profile of *K. pneumoniae* isolates (*n* = 12) tested against 16 antibiotics showed that these isolates were resistant to at least three antibiotics. In cecal isolates, *K. pneumoniae* showed resistance to all antibiotics tested, resulting in a MAR index equal to one. In contrast, stool isolates showed low resistance, with a MAR index equal to 0.187 for *K. oxytoca* (*n* = 7). High resistance was also observed in cecal isolates, which had a MAR index equal to one, while resistance in stool isolates was lower, with a MAR index equal to 0.125, as shown in Table 4.

### Prevalence of antibiotic resistance gene profiles

In this study, *K. pneumoniae* strains showed resistance against seven types of resistance genes, including tetracycline gene *tetA*, quinolone gene *gyrA*, β-lactamase genes *bla*_*TEM*_*, bla*_*CTX-M1*_, sulfonamides gene *sul1*, carbapenems gene *bla*_*OXA-48*_, peptides gene *mcr-1*, and macrolides gene *ermB* as shown in Table 5. The results showed that the highest positive resistance rate was for the macrolide gene *ermB* 11/12 (91.7%), followed by the β-lactamase genes *bla*_*TEM*_ 9/12 (75.0%) and *bla*_*CTX-M1*_ 8/12 (66.7%), the sulfonamides gene *sul1* 6/12 (50%) and the quinolone gene *gyrA* 5/12 (41.7%). On the other hand, the lowest positive rate was for *the* peptide gene *mcr-1*1/12 (8.3%), followed by the tetracycline gene *tetA* 2/12 (16.7%) and the carbapenemase gene *bla*_*OXA-48*_ 3/12 (25.0%). In chicken isolates, resistance was highest for the *ermB* gene (8/11), followed by *bla*_*TEM*_ (7/9), *bla*_*CTX-M1*_ (5/8), *sul1* (5/6), and *gyrA* (3/5). Resistance to *tetA* was observed in 2/2, while no isolates carried the *mcr-1* gene.

**Table 5:** Prevalence of genotypic detection of antibiotics resistance genes (*tetA, gyrA, bla*_*TEM*_*, bla*_*CTX-M1*_*, sul1, bla*_*OXA-48*_*, mcr-1*, and *ermB*) among *K. pneumonia* strains isolated from broiler chicken samples and human stool

Isolates source	Positive isolates for each resistance gene (%)							
	Tetracycline	Quinolone	β-lactamase		Sulfonamides	Carbapenem	Peptides	Macrolide
	*tetA*	*gyrA*	*bla*_*TEM*_	*bla*_*CTX-M1*_	*sul1*	*bla*_*OXA-48*_	*mcr-1*	*ermB*
Broiler chickens (*n* = 9)	2	3	7	5	5	2	0	8
Human (*n* = 3)	0	2	2	3	1	1	1	3
Total (*n* = 12)	2 (16.7)	5 (41.7)	9 (75.0)	8 (66.7)	6 (50.0)	3 (25.0)	1 (8.3)	11 (91.7)

In human isolates, 3/8 carried the *bla*_*CTX-M1*_ gene, 2/9 carried *bla*_*TEM*_, 3/11 were positive for *ermB*, and 1/6 carried *sul1*. In addition, 2/3 were positive for *gyrA*, and 1/2 for *mcr-1*, with no isolates carrying *tetA*.

**Fig. 2:**
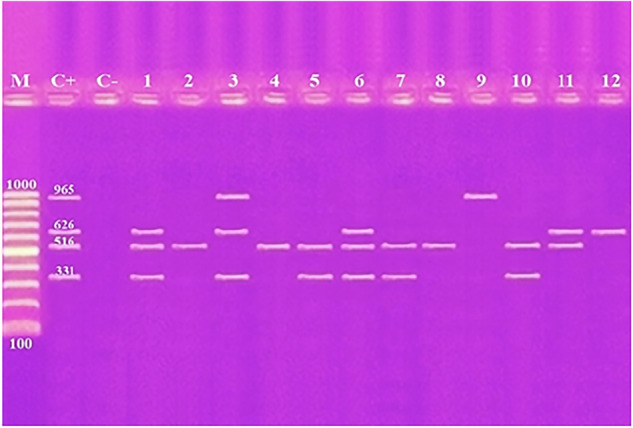
Agarose gel electrophoresis of multiplex PCR of *tetA* (965 bp), *gyrA* (626 bp), *bla*_*TEM*_ (516 bp) and *sul1* (331 bp) antibiotic resistance genes for characterization of *K. pneumoniae*. Lane M: 100 bp ladder as molecular size DNA marker. Lane C+: Control positive for *tetA*, *gyrA*, *bla*_*TEM*_ and *sul1* genes. Lane C−: Control negative. Lanes 3 & 9: Positive *K. pneumoniae* strains for *tetA* gene. Lanes 1, 3, 6, 11 & 12: Positive strains for *gyrA* gene. Lanes 1, 2, 4, 5, 6, 7, 8, 10 and 11: Positive strains for *bla*_*TEM*_ gene. Lanes 1, 3, 5, 6, 7 & 10: Positive strains for *sul1*gene. Lane from 1 to 9 for *K. pneumoniae* strains from broiler and Lane 10–12 for *K. pneumoniae* strains from human

**Fig. 3:**
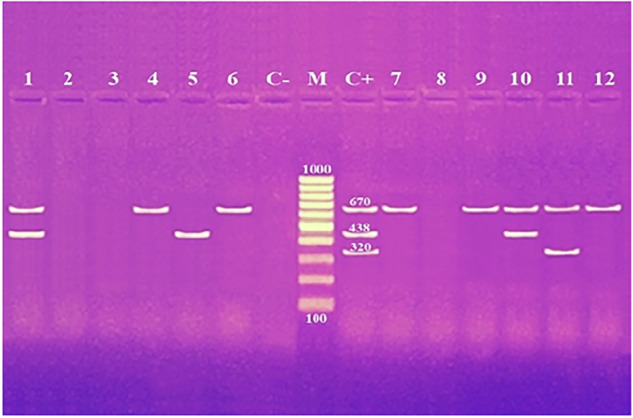
Agarose gel electrophoresis of multiplex PCR of *bla*_*CTX-M1*_ (670 bp), *bla*_*OXA-48*_ (438 bp) and *mcr-1* (320 bp) antibiotic resistance genes for characterization of *K. pneumoniae*. Lane M: 100 bp ladder as molecular size DNA marker. Lane C+: Control positive for *bla*_*CTX-M1*_, *bla*_*OXA-48*_ and *mcr-1* genes. Lane C−: Control negative. Lanes 1, 4, 6, 7, 9, 10, 11 and 12: Positive strains for *bla*_*CTX-M1*_ gene. Lanes 1, 5 & 10: Positive strains for *bla*_*OXA-48*_ gene. Lane 11: Positive strain for *mcr-1* gene. Lane from 1 to 9 for *K. pneumoniae* strains from broiler and Lane 10–12 for *K. pneumoniae* strains from human

**Fig. 4:**
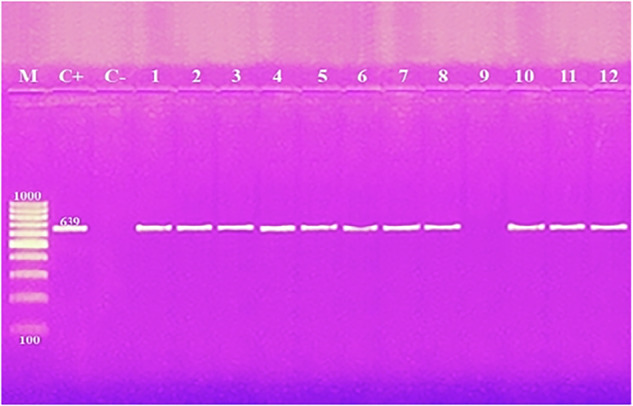
Amplification of *ermB* (639 bp) antibiotic resistance gene for characterization of *K. pneumoniae*. Lane M: 100 bp ladder as molecular size DNA marker. Lane C+: Control positive for *ermB* gene. Lane C−: Control negative.Lanes from 1 to 8, 10, 11 & 12: Positive strains for *ermB* gene. Lane 9: Negative strain for *ermB* gene. Lane from 1 to 9 for *K. pneumoniae* strains from broiler and Lane 10–12 for *K. pneumoniae* strains from human

Using primers for antibiotic resistance genes *tetA*, *gyrA*, *bla*_*TEM*_ and *sul1*, amplicons of length 965 bp, 626 bp, 516 bp, and 331 bp were amplified from two (16.7%), five (41.7%), eight (75.0%), and six (50.0%) of the 12 *K. pneumoniae* isolates examined from broiler and human samples, respectively (Fig. 2 and Table 5). Amplification of the antibiotic resistance genes *bla*_*CTX-M1*_, *bla*_*OXA-48*_ and *mcr-1* resulted in the detection of amplicons at 670 bp, 438 bp, and 320 bp from eight (66.7%), three (25.0%), and one (8.3%) of the *K. pneumoniae* isolates examined from broiler and human samples, respectively (Fig. 3 and Table 5). When investigating the *ermB* gene, a 639 base pair product was identified for 11 (91.7%) of the *K. pneumoniae* isolates tested (Fig. 4 and Table 5).

## Discussion

*Klebsiella* and other bacteria are serious pathogens that affect both humans and animals [2, 30]. The current study highlights that *K. pneumoniae* and *K. oxytoca* bacteria recovered from backyard broiler chickens and their human contacts exhibit variable MDR. Furthermore, the MDR of *K. pneumoniae* carrying multiple antimicrobial resistance genes was detected in samples from backyard broiler organs (cecum, liver, and thigh muscles) and stool samples from humans who had contact with them.

In the present study, *K. pneumoniae* was isolated from broiler chickens at a rate of 11.3% (9 out of 80 samples), which is higher than the isolation rate (7.08%) found in a poultry farm in India [31]. In turn, it was lower than the 25.8% recorded in Norway [32]. A previous report showed the presence of *K. pneumoniae* in 8% of lung and liver samples taken from apparently healthy chickens in Nigeria [33]. In a study conducted in Egypt [34], *Klebsiella* species were isolated from different organs of diseased chickens, with the highest recovery rate from the lungs (46.67%), followed by the liver and spleen, at 20%, and then the heart at 13.33%. It is generally known that *K. pneumoniae* is the primary pathogen of respiratory infections in poultry or can be associated with intestinal and systemic infections. In some cases, they may act as opportunistic secondary invaders, exacerbating existing clinical conditions [34, 35]. Our study was done to investigate the possible causes of clinical illness and to maximize the likelihood of isolating pathogenic strains associated with disease. While including healthy birds, as previously described [33], would provide important information on asymptomatic carriers, our intention in this preliminary work was to concentrate on clinically affected birds.

In this study, cecal samples showed the highest detection rate of 35%, followed by liver and thigh muscles (5% each). This may be due to the fact that the anaerobic environment of the cecum promotes the growth of facultative anaerobic bacteria such as *Klebsiella* [36]. Contamination of poultry meat can occur during processing, especially during the evisceration stage, when cecal contents can transfer these microorganisms to the surface of the carcass, increasing the risk of foodborne illness [37]. Importantly, the heart samples did not show the presence of *K. pneumoniae* bacteria. Previous studies reported in Egypt showed a low rate of *K. pneumoniae* infection in broiler livers of 2.5% [2]. The prevalence of *K. pneumoniae* in chicken thigh muscles is relatively low, with only 5% of samples showing positive results. This contrasts sharply with a study by researchers [38] who found a rate of 60% (high prevalence) after analyzing chicken meat samples from stores in five countries in Europe. Among these countries, Denmark recorded the highest prevalence of *K. pneumoniae* in meat samples collected from different countries in Denmark, with 90%, followed by France with 73% [39]. These findings emphasize the potential importance of food in the colonization and infection of *K. pneumoniae* bacteria in humans [38]. The prevalence of *K. pneumoniae* in chicken cloacal swabs demonstrates that this bacterium is firmly established in the gastrointestinal tract of chickens, posing a serious risk of carcass contamination and potential human infection [40].

In stool samples from individuals who had contact with birds, the detection rate was 15% (3 of 20), which is much higher than the 0.5% found in outpatients in China [41], and contrasts with the 38% previously documented [42]. Furthermore, *K. pneumoniae* bacteria have been detected in the feces of farm workers and veterinarians [5].

In the present study, the prevalence of *K. oxytoca* in broiler chickens was 3/80 (3.75%), while 20% (4/20) were positive in human stool. In Egypt, 7/28 (25%) of *K. oxytoca* was isolated from chicken organs [43]. Smith et al. isolated *K. oxytoca* from 14 of 200 (7.0%) human patients in Halifax, Nova Scotia [44].

The string test was performed only for *K. pneumoniae* because hypermucoviscosity, which the test is designed to detect, is a recognized virulence phenotype primarily associated with this species [17, 18]. Moreover, the main objective of our study was to investigate *K. pneumoniae* isolates, and therefore, additional phenotypic tests were not applied to *K. oxytoca*. In the current study, with regard to the string test results of *K. pneumoniae* isolates, 7 isolates were classified as hvKp, which included 5 isolates from the cecum and 2 isolates from human stool. The remaining five isolates were identified as cKp. Both pathotypes of *K. pneumoniae* are found worldwide; however, hvKp is more harmful than cKp [45]. Over the past three decades, the prevalence of hvKp infection has gradually increased in the Asia-Pacific region, often affecting healthy individuals in the community [46]. Research highlights that *K. pneumoniae* infection can lead to serious conditions, such as central nervous system infection and endophthalmitis [47]. In contrast, cKp is the most common infectious agent in Western countries, where it typically acts as an opportunistic pathogen [48]. This pathogen primarily infects immunocompromised individuals, especially in healthcare settings [49].

The capsule is an essential virulence component that protects *K. pneumoniae* from phagocytosis and lethal agents in serum. K1 and K2 are the two most important serotypes, have been confirmed to be pathogenic in mice, and are commonly involved in community-acquired pneumonia [50]. According to this study, the most common serotype in humans and broilers was capsular K1 (83%, 10/12).

In this study, antimicrobial susceptibility testing showed that all tested *Klebsiella* isolates (*n* = 19) were resistant to erythromycin and clindamycin (100%). Resistance rates were 94.7% to penicillin and 84.2% to ampicillin, while only 10.5% showed resistance to colistin and ofloxacin. Abd ElGawad et al. found that all tested isolates (*n* = 20) showed resistance to ampicillin and oxytetracycline [2]. Furthermore, 90% (18/20) of the isolates exhibited erythromycin resistance. In a study conducted in Iraq, *K. oxytoca* was isolated from urine samples and found to be sensitive to ciprofloxacin. However, it showed resistance to amoxicillin and cefotaxime [51].

The rising numbers of MDR bacteria and the potential risk of zoonotic bacterial spreading from animals to humans are important issues that we need to address [52]. The lack of effective vaccines and environmentally friendly prevention methods makes it necessary to treat foodborne bacterial infections of zoonotic origin with antibiotics. The overuse of antibiotics in animals and poultry has led to an increase in the number of MDR bacteria, posing a threat to human and animal health worldwide [53].

The presence of resistance genes is one of the reasons for bacterial resistance. *K. pneumoniae* bacteria have various mechanisms for antimicrobial agent resistance, and the prevalence of resistant *K. pneumoniae* bacteria is increasing, making the treatment of infections caused by these bacteria more difficult. In the current study, *K. pneumoniae* isolates showed significant resistance to macrolide genes, especially *ermB* (91.7%). In addition, significant resistance was observed against β-lactamase genes, with *bla*_*TEM*_ at 75% and *bla*_*CTX-M1*_ at 66.7%. The sulfonamide gene *sul1* showed a remarkable resistance rate of 50%, while the quinolone gene *gyrA* showed a resistance rate of 41.7%. Furthermore, 25% of the isolates exhibited resistance to the carbapenem gene *bla*_*OXA-48*_. In contrast, resistance was low for the tetracycline gene *tetA* at 16.7% and the peptide gene *mcr-1* at 8.3%. In a recent study, *K. pneumoniae* bacteria were isolated from broiler chickens on Chinese farms. The researchers reported that 100% of the isolates contained β-lactam resistance genes, with only one isolate showing resistance to carbapenem and peptide genes [53]. In comparison, the ESBL production rate in a healthy Indian meat and poultry farm was 5% (1 in 20) [31]. In Indonesia [14], 20% of cloacal swab samples collected from broiler farms tested positive for *K. pneumoniae*. All isolates contained the *gyr*A and bla_*TEM*_ genes, with 85% also containing the *tetA* gene and 53% the *ermB* gene. In a study conducted in Kenya, researchers recovered 87 isolates of *K. pneumoniae* and three isolates of *K. oxytoca from* human stool samples. These isolates were tested for antibiotic resistance genes across different classes. The results showed that the *bla*_*TEM*_ gene was resistant in 58% of the isolates, 9% for the *bla*_*CXT-M1*_ gene, 18% for the *tetA* gene, and 31% for the *sul1* gene [54]. In our study, the rate of macrolide resistance was relatively high (91.7%), which may be explained by the frequent use of macrolides in poultry farms, either for prophylaxis or treatment, and in some cases as growth promoters in poultry production. Such practices exert selective pressure that facilitates the emergence and persistence of resistant strains.

In our study, we did not conclude that direct transmission from chickens to humans occurred; rather, we reported the isolation of similar organisms from both sources. This raises the possibility of zoonotic transmission. Moreover, we isolated the same serotypes from poultry and human samples, and these isolates harbored resistance genes, further supporting the potential for cross-species dissemination of resistant strains. Actually, the number of human samples was small; however, these represented the only available samples from individuals who were in direct contact with chickens. While this limits the strength of epidemiological conclusions, our intention was not to make broad generalizations but rather to provide preliminary data highlighting the presence of the pathogen in both humans and chickens. In addition, the collection of human fecal samples poses practical and ethical challenges, which further contributed to the limited sample size. The primary goal was to establish baseline data regarding the occurrence and antimicrobial resistance of isolates from humans and chickens. The incorporating genomic analysis would reinforce these findings and intend to pursue this in future research.

Finally, *Klebsiella* species were detected in 19% of samples, with *K. pneumoniae* isolated in 12% and *K. oxytoca* in 7%. *K. pneumoniae* showed a prevalence of 11.25% in broiler chickens and 15% in human contacts, with serotype K1 dominating both. All isolates were fully resistant to erythromycin and clindamycin but showed low resistance to colistin (10.5%). The *ermB* gene was present in 91.6%, along with genes for resistance to β-lactams, sulfonamides, quinolones, and carbapenems. The detection of *K. pneumoniae* in broiler chicken samples represents a significant public health threat, especially for immunocompromised individuals, severely complicating treatment options. Thus, further studies are needed on *K. pneumonia*e isolates from broiler chickens and humans, including serotyping and innovative solutions to escape antibiotic resistance. It is important to pay attention to improving the level of biosecurity in backyards, which is crucial to preventing human infection with zoonotic poultry diseases resulting from human-animal interactions.

## Conclusion

There was a high prevalence of MDR *K. pneumoniae* and *K. oxytoca* in backyard broiler chickens and their contacts with *K. pneumoniae* AMR genes in Egypt. *Klebsiella* species were found in a significant number of samples, with *K. pneumoniae* being more prevalent than *K. oxytoca. K. pneumoniae* has been identified in both broiler chickens and their human contacts, with serotype K1 being the most common in both. All isolates showed complete resistance to erythromycin and clindamycin, while resistance to colistin was relatively low. The *ermB* gene has been commonly identified, along with several resistance genes to β-lactams, sulfonamides, quinolones, and carbapenems. The spread of *K. pneumoniae* bacteria in broiler chickens is a major public health concern, especially for immunocompromised humans. This situation underscores the need for further research on *K. pneumoniae* isolates from poultry and humans, with a focus on developing effective strategies such as vaccination, the use of probiotics, enhancement of biosecurity and farming practices, and the exploration of novel antimicrobial agents like bacteriophages and antimicrobial peptides, to curb antibiotic resistance and prevent zoonotic disease transmission.
